# Prophylaxis against spontaneous bacterial peritonitis: Too much or too little?

**DOI:** 10.1017/ash.2022.232

**Published:** 2022-06-17

**Authors:** Muhammad Dhanani, Aparna Krishnamoorthy, Jody D. Ciolino, Kalpana Gupta, Judith M. Strymish

**Affiliations:** 1 Division of Infectious Diseases, Department of Medicine, Feinberg School of Medicine, Northwestern University, Chicago, Illinois; 2 Medical Service, Veterans Affairs Boston Healthcare System, Boston, Massachusetts; 3 Section of Infectious Diseases, Department of Medicine, Boston Medical Center, Boston, Massachusetts; 4 Institute for Public Health and Medicine, Feinberg School of Medicine, Northwestern University, Chicago, Illinois; 5 Division of Biostatistics, Department of Preventive Medicine, Feinberg School of Medicine, Northwestern University, Chicago, Illinois; 6 Section of Infectious Diseases, Department of Medicine, Boston University School of Medicine, Boston, Massachusetts; 7 Department of Medicine, Harvard Medical School, Boston, Massachusetts

## Abstract

Prophylaxis against spontaneous bacterial peritonitis (SBP) is recommended for select patients with cirrhosis, but long-term antibiotic therapy has risks. We evaluated concordance with guideline recommendations in 179 veterans with cirrhosis; 55% received guideline-concordant management of SBP prophylaxis. Despite stable guideline recommendations since 2012, guideline adherence remains low.

Prophylaxis against spontaneous bacterial peritonitis (SBP) improves mortality rates among patients with cirrhosis and hypoproteinemic ascites.^1,2^ In 2012, the American Association for the Study of Liver Disease (AASLD) published clinical practice guidelines recommending daily norfloxacin or trimethoprim-sulfamethoxazole (TMP-SMX) for patients with cirrhosis, hypoproteinemic ascites and renal or hepatic dysfunction.^3^ A 2021 update to these recommendations acknowledged the role of ciprofloxacin due to the unavailability of norfloxacin.^4^

However, inappropriate long-term antibiotic use has recognized risks. Patients receiving SBP prophylaxis at are at risk of developing drug-resistant infections,^5^ prompting calls for stewardship.^6^ Actual concordance with published guidelines is unknown and is relevant given the risks associated with prolonged antibiotic use. In this retrospective study, we assessed rates of concordance with guideline-recommended management among a cohort of veterans.

## Methods

In this retrospective cohort study, all patients undergoing paracentesis at the Boston Veterans Affairs Medical Center between January 1, 2014, and December 31, 2018, were screened for inclusion. Exclusion criteria included absence of cirrhosis and hepatic transplantation. Additionally, patients aged ≥90 years were excluded pursuant to a data use agreement. Demographic parameters including receipt of primary care at the medical center or an outlying clinic, gastroenterology involvement, and prescription data were recorded manually.

The start date of the 1-year observation period was determined in a cascading fashion. First, we used the date of the first outpatient prescription for SBP prophylaxis. If no such prescriptions existed, we used the date when peritoneal fluid studies were consistent with SBP, bacterascites, or culture-negative neutrocytic ascites. If fluid studies were not consistent with these conditions at any point, we defined the start date as the date when peritoneal fluid studies met guideline criteria for SBP prophylaxis utilization. If none of these conditions was met, we defined the start date as the date of the first paracentesis within the eligibility window.

Guideline criteria for SBP prophylaxis initiation comprise (1) peritoneal fluid protein level <1.5 g/dL present along with serum sodium level ≤130 mEq/L, blood urea nitrogen level ≥25 mg/dL, or serum creatinine level ≥1.2 mg/dL, or (2) peritoneal fluid protein level <1.5 g/dL present along with serum total bilirubin level ≥3 mg/dL. The Child-Pugh score component of the criteria was not incorporated because of expected inconsistency in documentation of its parameters.

After application of inclusion and exclusion criteria, patients were classified as receiving guideline-concordant care if they received SBP prophylaxis upon meeting the guideline-recommended criteria, or if it was appropriately withheld. Patients prescribed indicated SBP prophylaxis that deviated from the guideline-recommended dosing frequency were classified as having received guideline-discordant care. Patients initially receiving guideline-discordant care who later received guideline-concordant care were kept in the guideline-discordant group. The Institutional Review Board at the Veterans Affairs Boston Healthcare System approved all study protocols.

Descriptive statistics summarized patient characteristics. To explore and quantify associations between patient characteristics and receipt of guideline-concordant care, we first used a series of unadjusted logistic regression models with concordance as the outcome. We then used purposeful model selection to develop an adjusted, multiple logistic regression model. The statistical analysis was performed using SAS version 9.4 software (SAS Institute, Cary, NC). Significance was set at a 2-sided 0.05 α level.

## Results

Of 259 patients identified for possible inclusion, 179 patients (69.1%) met eligibility criteria. The most common reasons for exclusion were absence of cirrhosis (65 patients; 25.1%), hepatic transplantation (5 patients, 1.9%) and age ≥90 years (4 patients, 1.5%). The median age of the cohort was 64.0 years (interquartile range [IQR], 57.9–68.4) and 99.4% were men. Prior to the end of the observation period, 101 patients (56.4%) died. Over the year preceding the observation period, gastroenterology involvement was documented among 122 patients (68.2%), with 91 (50.8%) having been seen as outpatients and 88 (49.2%) seen exclusively while hospitalized. During the observation period, inpatient gastroenterology consultation was completed for 96 patients (53.6%) (Table 1).

**Table 1.** Characteristics of Cohort, Grouped by Type of Care Received (N = 179)

	Overall	Guideline Concordant	Guideline Discordant
Variable	(N=179)	(N=99)	(N=80)
Age, median y [IQR]	64.01 [57.94–68.44]	63.87 [58.97–68.98]	64.39 [57.17–68.16]
**Race, no. (%)**			
White	162 (90.5)	87 (87.9)	75 (93.8)
Nonwhite^a^	8 (4.5)	8 (8.1)	0 (0.0)
Declined/Refused	9 (5.0)	4 (4.0)	5 (6.2)
**Death during observation, no. (%)**			
No	78 (43.6)	47 (47.5)	31 (38.8)
Yes	101 (56.4)	52 (52.5)	49 (61.3)
Time in cohort, median d [IQR]	264 [45–365]	307 [84–365]	161 [34–365]
**Site of primary care, no. (%)**			
PCP at VABHS	92 (51.4)	56 (56.6)	36 (45.0)
PCP outside VABHS	87 (48.6)	43 (43.4)	44 (55.0)
**Any GI clinic visit over observation, no. (%)**			
Not seen by GI	84 (46.9)	44 (44.4)	40 (50.0)
Seen by GI	95 (53.1)	55 (55.6)	40 (50.0)
**Any GI ward visit over observation, no. (%)**			
Not seen by GI	83 (46.4)	55 (55.6)	28 (35.0)
Seen by GI	96 (53.6)	44 (44.4)	52 (65.0)
**Any GI visit over prior year, no. (%)**			
Not seen by GI	57 (31.8)	33 (33.3)	24 (30.0)
Seen by GI	122 (68.2)	66 (66.7)	56 (70.0)
**GI visit over prior year, ward only, no. (%)**			
Not seen by GI exclusively as inpatient	148 (82.7)	84 (84.8)	64 (80.0)
Seen by GI exclusively as inpatient	31 (17.3)	15 (15.2)	16 (20.0)
**GI visit over prior year, clinic only, no. (%)**			
Not seen by GI exclusively as outpatient	88 (49.2)	48.5 (48.5)	40 (50.0)
Seen by GI exclusively as outpatient	91 (50.8)	51 (51.5)	40 (50.0)

Note. PCP, primary care physician; VABHS, Veterans Affairs Boston Healthcare System; GI, gastroenterology; IQR, interquartile range.

a Black, African-American, Native American, or Pacific Islander.

Guideline-concordant management of SBP prophylaxis was observed in 99 patients (55.3%). Among 93 patients meriting antibiotics, 65 (70.0%) did not receive them. Conversely, among 86 who did not have an indication for antibiotic prophylaxis, 15 (17.4%) received it.

Gastroenterological evaluation over the year preceding the observation period occurred among 66 patients in the guideline-concordant group (66.7%) and 56 patients (70.0%) of the guideline-discordant group (OR, 0.86; 95% CI, 0.45–1.62; *P* = .63). Inpatient gastroenterology evaluation during the observation period was associated with lower odds of receiving guideline-concordant care (OR, 0.43; 95% CI, 0.24–0.79; *P* < .01), a finding supported by multiple regression results (aOR, 0.44; 95% CI, 0.24–0.81; *P* < .01). Gastroenterology evaluation limited to the outpatient setting during the observation period did not have such an association (OR, 1.25; 95% CI, 0.69–2.26; *P* = .46) (Table 2).

**Table 2. A.** Univariate Associations Between Patient Characteristics and Guideline Concordance

Model	Variable	Odds Ratio	95% CI	*P* Value
1	Received prophylaxis	1.709	0.839–3.482	.14
2	Race, nonwhite^a^	…	…	.02
3	Race, declined	…	…	…
4	Age	1.015	0.981, 1.049	.39
5	PCP outside VABHS	0.628	0.347, 1.137	.13
6	Any GI clinic visit over observation	1.250	0.692, 2.257	.46
7	Any GI ward visit over observation	0.431	0.235, 0.790	.01
8	Death occurred	0.700	0.385, 1.273	.24
9	Any GI visit over prior year	0.857	0.454, 1.617	.63
10	GI visit over prior year, ward only	0.714	0.329, 1.552	.40
11	GI visit over prior year, clinic only	1.062	0.589, 1.916	.84

Note. CI, confidence interval; PCP, primary care physician; VABHS, Veterans Affairs Boston Healthcare System; GI, gastroenterology; IQR, interquartile range.

a *P* value corresponds to Fisher exact test due to zero counts.

Nonwhite patients, all of whom self-identified as Black, African-American, Native American, or Pacific Islander, had a lower odds of receiving guideline-concordant care with *P* < .05 by the Fisher exact test. Due to low cell counts and model instability, we were unable to include race in the multiple logistic regression (Table 2). Other factors, such as age and site of primary care, were not significantly associated with the odds of receiving guideline-concordant care in this data set.

**Table 2. B.** Multiple Variable Associations Between Patient Characteristics and Guideline Concordance

Variable	Odds Ratio	95% CI	*P* Value
Race, nonwhite^a^	N/A	N/A	N/A
Race, declined	0.621	0.156–2.472	.50
Any GI ward visit over observation	0.436	0.235–0.811	.01

Note. CI, confidence interval; GI, gastroenterology.

a Not estimable due to zero cell counts (reference category: race, white).

Ciprofloxacin was used in 26 patients (14.5%) and 12 patients (6.7%) received TMP-SMX; 4 patients (2.2%) received a mixture of these 2 agents. One patient received norfloxacin.

## Discussion

Just over half of the cohort received guideline-concordant management of SBP prophylaxis. Underutilization of SBP prophylaxis was observed in 70% of the cohort. Moreover, ∼17% of the cohort received SBP prophylaxis without a guideline-supported indication. Receipt of guideline-concordant care was not more common among patients seen by gastroenterology specialists.

Nonadherence to published guideline recommendations is common. In one systematic review, guideline adherence ranged between 34.7% and 91.8%.^7^ A study of adherence to the clinical practice guideline for management of hepatic encephalopathy showed that 22% of patients underwent a guideline-concordant diagnostic evaluation.^8^

Although this study did not evaluate reasons for guideline nonadherence, it is possible that guideline nonadherence reflected the prescriber’s skepticism of the guideline recommendations given the mixed results of studies evaluating the impact of SBP prophylaxis.^9,10^ Gastroenterology involvement may increase scrutiny of the risk–benefit balance of prophylaxis and factor in additional nuances of complicated cases, reducing strict adherence with the algorithmic approach of a guideline. Notably, the agents of choice for prophylaxis (fluoroquinolones) have prominent adverse effects.

This study had several limitations. In this study, we captured a 5-year period beginning about a year after publication of the guideline recommendations; those recommendations may have been too new to demonstrably impact clinical practice over that time frame. The retrospective design could result in misclassification bias. Factors used to define the cohort could not be analyzed as covariates but may have been relevant to clinical decision making. Due to the small sample size and the exploratory nature of the analyses, there were no formal power calculations. Evaluation of healthcare utilization and mortality could not be performed because comorbid conditions were not assessed. Finally, the male predominance of the study cohort and the inclusion of patients only from 1 site may diminish the generalizability of these results.

Concordance with published guidelines regarding SBP prophylaxis remains low. Strategies to improve clinicians’ adherence to these recommendations are needed.

## References

[1] Facciorusso A , Papagiouvanni I , Cela M , Buccino VR , Sacco R. Comparative efficacy of long-term antibiotic treatments in the primary prophylaxis of spontaneous bacterial peritonitis. Liver Int 2019;39:1448–1458. 30920712 10.1111/liv.14109

[2] Wang W , Yang J , Liu C , et al. Norfloxacin, ciprofloxacin, trimethoprim-sulfamethoxazole, and rifaximin for the prevention of spontaneous bacterial peritonitis: a network meta-analysis. Eur J Gastroenterol Hepatol 2019;31:905–910. 31107737 10.1097/MEG.0000000000001446

[3] Runyon BA. Introduction to the revised American Association for the Study of Liver Diseases practice guideline management of adult patients with ascites due to cirrhosis 2012. Hepatology 2013;57:1651–1653. 10.1002/hep.26359 23463403

[4] Biggins SW , Angeli P , Garcia-Tsao G , et al. Diagnosis, evaluation, and management of ascites, spontaneous bacterial peritonitis and hepatorenal syndrome: 2021 practice guidance by the American Association for the Study of Liver Diseases. Hepatology 2021;74:1014–1048. 33942342 10.1002/hep.31884

[5] Fernandez J , Acevedo J , Castro M , et al. Prevalence and risk factors of infections by multiresistant bacteria in cirrhosis: a prospective study. Hepatology 2012;55:1551–1561. 22183941 10.1002/hep.25532

[6] Gonzalez SA. Antibiotic prophylaxis for spontaneous bacterial peritonitis: benefit or risk? Am J Gastroenterol 2019; 114:553–555. 30920419 10.14309/ajg.0000000000000208

[7] Arts DL , Voncken AG , Medlock S , Abu-Hanna A , van Weert HCPM. Reasons for international guideline nonadherence: a systematic review. Int J Med Inform 2016;89:55–62. 26980359 10.1016/j.ijmedinf.2016.02.009

[8] Kumral D , Qayyum R , Roseff S , Sterling RK , Siddiqui MS. Adherence to recommended inpatient hepatic encephalopathy workup. J Hosp Med 2019;14:157–160. 30811321 10.12788/jhm.3152

[9] Bajaj JS , Tandon P , O’Leary JG , et al. Outcomes in patients with cirrhosis on primary compared to secondary prophylaxis for spontaneous bacterial peritonitis. Am J Gastroenterol 2019;114:599–606. 30694868 10.14309/ajg.0000000000000044 PMC6450703

[10] Komolafe O , Roberts D , Freeman SC , et al. Antibiotic prophylaxis to prevent spontaneous bacterial peritonitis in people with liver cirrhosis: a network meta-analysis. Cochrane Database Syst Rev 2020;1(1):CD013125. 31978256 10.1002/14651858.CD013125.pub2 PMC6984637

